# Buyang Huanwu Tang inhibits cellular epithelial-to-mesenchymal transition by inhibiting TGF-β1 activation of PI3K/Akt signaling pathway in pulmonary fibrosis model in vitro

**DOI:** 10.1186/s12906-019-2807-y

**Published:** 2020-01-15

**Authors:** Zi-fei Yin, Yang-lin Wei, Xuan Wang, Li-na Wang, Xia Li

**Affiliations:** 1 Department of Traditional Chinese Medicine, Changhai Hospital, The Second Military Medical University, No.168 Changhai Road, Shanghai, 200433 China; 2 Seventh People’s Hospital of Shanghai University of Traditional Chinese Medicine, No.358 Datong Road, Shanghai, 200137 China

**Keywords:** Pulmonary fibrosis, Buyang Huanwu Tang, Akt, PI3K, A549 cells

## Abstract

**Background:**

Pulmonary fibrosis (PF) is a chronic and progressive interstitial lung disease. Buyang Huanwu Tang (BYHWT), a classical traditional Chinese medicine formula, has been widely utilized for the treatment of PF in China. This present study aimed to explore the mechanism of BYHWT in the treatment of PF in vitro.

**Methods:**

TGF-β1 stimulated human alveolar epithelial A549 cells were used as in vitro model for PF. Post the treatment of BYHWT, cell viability was measured by MTT assay, and cell morphology was observed under microscope. The epithelial-to-mesenchymal transition (EMT) markers (E-cadherin, Vimentin) and collagen I (Col I) were detected by western blot, immunofluorescence staining and real-time quantitative polymerase chain reaction. With the co-administration of activators (IGF-1, SC79) and inhibitors (LY294002, MK2206), the effect of BYHWT on PI3K/Akt pathway was analyzed by western blot.

**Results:**

BYHWT inhibited cell growth, and prevented cell morphology changed from epithelial to fibroblasts in TGF-β1 induced A549 cells. BYHWT decreased Vimentin and Col I, while increased E-cadherin at both protein and mRNA levels. Moreover, phosphorylation of PI3K (p-PI3K) and phosphorylation of Akt (p-Akt) were significantly down-regulated by BYHWT in TGF-β1 stimulated A549 cells.

**Conclusion:**

These results indicate that BYHWT suppressed TGF-β1-induced collagen accumulation and EMT of A549 cells by inhibiting the PI3K/Akt signaling pathway. These findings suggest that BYHWT may have potential for the treatment of PF.

## Background

Pulmonary fibrosis (PF) is defined as a progressive lung injury disease with unknown pathogeny, which is characterized by aberrant proliferation of fibroblasts, damage of alveolar epithelial cell, and excessive collagen accumulation [1, 2]. The 4-year risk of death with PF is almost 41.0% [3]. New drugs including pirfenidone, nintedanib are not potential in alleviating PF. The prognosis of PF is overall poor, with the mortality rate ranged from 30 to 40% [4]. Thus, it is urgently needed to identify new potential therapeutic agents for PF patients.

The common feature of PF includes massive matrix extracellular deposition and excessive collagen accumulation [5]. The transforming growth factor (TGF)-β1 was recognized as the most important cytokine for fibrosis and was demonstrated to be a master inducer of epithelial-to-mesenchymal transition (EMT) in normal alveolar epithelial cells (AECs) [6, 7]. In addition to direct production of myofibroblasts, EMT can also indirectly create a profibrotic microenvironment by releasing cytokines [8]. As a result, the epithelial cell phenotype such as E-cadherin was lost and transformed into a mesenchymal cell phenotype such as Vimentin [9]. This stimulates alveolar EMT in AECs and trans-differentiation of resting fibroblasts to myofibroblasts, which leads to excessive production of fibrous collagen [10].

Previous investigations have demonstrated that the activation of PI3K/Akt signaling pathway induced by TGF-β1 is a key step in the development of PF, resulting in EMT, fibroblast proliferation and collagen accumulation [11, 12], whereas blocking PI3K/Akt signaling pathway ameliorates PF in animal models [13], indicating that pharmacological inhibition of PI3K/Akt signaling pathway might be a potential treatment of PF.

Bu-yang Huan-wu Tang (BYHWT) was originally described in the traditional Chinese medicine literature of Yi-Lin-Gai-Guo written by Qing-Ren Wang in 1830 during the Qing Dynasty. Based on Chinese medicine theory, BYHWT can enhance blood circulation and activate energy (qi) flow through energy meridians. Thus far, BYHWT has been prescribed for PF patients in China with promising outcome [14, 15]. However, the underlying mechanism remains unknown. Due to the vital importance of TGF-β1 mediated activation of PI3K/Akt pathway in the development PF, we preformed these experiments to elucidate the mechanism for the anti-PF effect of BYHWT.

## Methods

### Reagents

Transforming growth factor beta1 (TGF-β1, Cat:AF-100-21C) and Insulin-like growth factor-1(IGF-1, Cat:100–11-100) were purchased from PeproTech. MTT (3-(4,5-dimethylthiazol-2-yl)-2,5-diphenyl tetrazolium bromide, Cat:A21051) was purchased from Beyotime Biotechnology (ShangHai, China). SC79 (Cat: S7863), MK2206 (Cat: S1078), and LY294002 (Cat: S1105) were purchased from Selleck (ShangHai, China).

### Preparation of Buyang Huanwu Tang (BYHWT)

The constituents of BYHWT used as follows: Astragalus membranaceus (60 g), Radix Paeoniae Rubra (18 g), Rhizoma Ligustici Wallichii (9 g), Angelica sinensis (18 g), Pheretima aspergillum (9 g), Amygdalus persica (9 g), and *Carthamus tinctorius* (9 g). All the components were purchased from Changhai Hospital of Shanghai. In preparing BYHWT, the mixture of the components was soaked in distilled water for 30 min and then boiled in 8 volumes of water (v/w) for 1 h and extracted twice. This preparation method was the same to clinical preparation. The extract was centrifuged at 6000×g for 20 min and then the supernatant solution was condensed to concentration of 1 g/ml by water bath. The concentration of BYHWT was expressed in total dry weight of the crude herbs per milliliter in decoction. BYHWT was stored in − 20 °C.

### Cell culture and treatment

Human normal alveolar epithelial A549 (Cat:SCSP-503) cells were purchased from the Cell Bank of the Chinese Academy of Sciences (Shanghai, China), and cultured in Dulbecco’s Modified Eagle’s Medium (DMEM) (HyClone, USA) supplemented with 10% fetal bovine serum (FBS, Gibco, USA) and a 1% antibiotic solution (100 U/ml penicillin and 0.1 mg/ml streptomycin) at 37 °C with 5% CO2. After 24 h of serum starvation, 10 ng/ml TGF-β1 was supplemented to induce lung fibroblast activation and EMT.

### MTT assay for cell viability

Cells were seeded in 96-well plates with a density of 5000 cells/well. After 24 h of serum starvation, 10 ng/ml TGF-β1 with or without various concentrations of BYHWT (0.1, 0.5, 1, 5, 10, 50, 100 mg/ml) were administrated to cells for 24 h, 48 h, and 72 h, respectively. Then, cell medium was discarded and each well was added with 100 μl of DMEM medium containing 10% MTT (5 mg/ml). Four hours later, each well was added with 100 μl dissolved solution (10% SDS, 5% isobutanol, 0.012 mol/L HCL). After being incubated overnight, the absorbance at a wavelength of 570 nm was measured using a multiskan spectrum microplate reader. The cell viability was calculated by the following formula: cell viability = (A of the control group-A of the experimental group) / (A of the control group-A of the blank group) × 100%. All the experiments were repeated at least three times.

### Western blot

Cells were seeded in 6-well plates, followed by the administration of TGF-β1 or PI3K/Akt pathway activators and inhibitors with or without BYHWT for the indicated time. Cells was washed by Phosphate Buffered Saline (PBS, Hyclone), and then the whole cell proteins were extracted by the cell-lysis mixture including RIPA (Beyotime), PMSF (Beyotime) and protease inhibitors cocktail (Roche). The extracted proteins were quantified by BCA protein assay kit (Pierce, Thermo Scientific) and then loaded for 10% SDS-PAGE gel electrophoresis, transferred to PVDF membranes (Millipore), and blocked in 5% milk. Next, the membranes were incubated with the primary antibodies overnight at 4 °C and subsequently the HRP-conjugated secondary antibodies (CST, 7076; CST 7074) for 2 h at room temperature. Human β-actin (CST, 3700) was used as a loading control. Primary antibodies were used as follows: E-cadherin (CST, 3195), Vimentin (CST, 5741), Col-I (Santa Cruz, sc-293,182), PI3K (CST, 4257), phosphorylated PI3K (CST, 4228), AKT (CST, 4691) and phosphorylated AKT (CST, 4058). Membranes were washed and visualized using enhanced chemiluminescence kit (Pierce, Thermo) and Gel Imaging System (Syngene).

### Immunofluorescence staining

For immunofluorescence, cells were washed with PBS and fixed in methanol for 20 min at 37 °C. Then cells were blocked by 5% BSA (bovine serum albumin) for 1 h at 37 °C. Next, cells were incubated with primary antibodies (E-cadherin, Vimentin, and Col-I) diluted in 5% BSA (1:200) at 4 °C overnight. Then, cells were washed thrice with PBS and incubated with FITC or PE-conjugated secondary antibodies for 1 h at 37 °C. Cells were incubated with 4′, 6-diamidino-2-phenylindole (DAPI) for 20 min at room temperature, followed by the observation by a fluorescence microscope.

### RNA extraction and quantitative real-time PCR (RT-qPCR)

Total RNA was isolated from A549 cells with Trizol Reagent (Invitrogen), followed by quantification with the Nanodrop Spectrophotometer (Thermo Scientific). RNA samples were performed for first-strand cDNA synthesis by Primescript RT Master Mix (Takara) under thermocycler (Applied Biosystems). The cDNA level was measured by Real-time qPCR (Applied Biosystems) that was run with SYBR-Green master mix (TOYOBO). Each sample was examined in triplicate and the mean values were used for calculation. Specificity of the amplified PCR products was determined by melting curve analysis. The following primers for the amplification of the genes were used: 5′-TAGGGTCTAGACATGTTCAGCTTTGT-3′ (ColI forward), and 5′-GTGATTGGTGGGATGTCTTCGT-3′ (ColI reverse); 5′-AGAGGAAGCCGAAAACACCC-3′ (Vimentin forward), and 5′-GGCTTGGAAACATCCACATCG-3′ (Vimentin reverse); 5′-ATGCTGATGCCCCCAATACC-3′ (E-cadherin forward), and 5′-TACTGCTGCTTGGCCTCAAA-3′ (E-cadherin reverse) ; 5′-CGAGGCCCAGAGCAAGAG-3′ (β-actin forward), and 5′-CCACACGCAGCTCATTGTA-3′ (β-actin reverse). Relative gene expression levels were calculated using the formula 2^−ΔΔCT^.

### Statistical analysis

All data was presented as mean ± standard error from three separate experiments performed. Statistical analysis was performed using SPSS 20.0 software (SPSS, Chicago, IL). One-way analysis of variance was used to compare the difference between groups. Differences were considered significant if *P* < 0.05.

## Results

### Inhibitory activity of BYWHT in TGF-β1 stimulated A549 cells

**Fig. 1:**
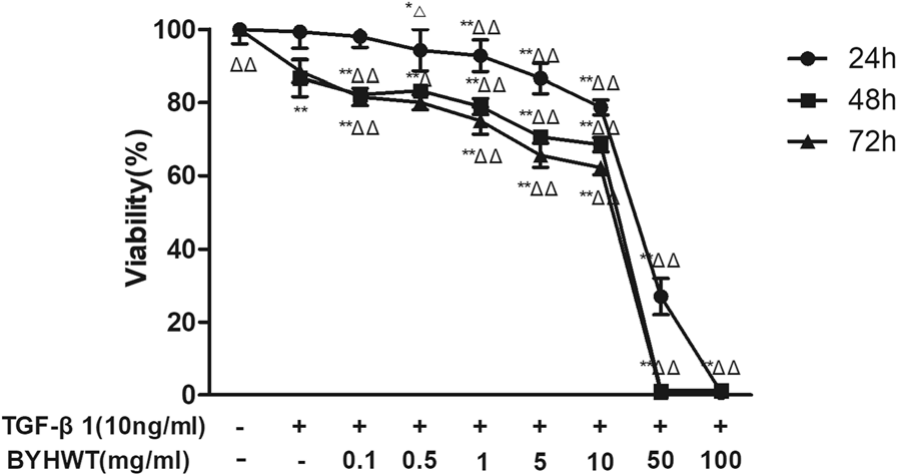
Inhibitory activity of BYHWT on the A549 cells by the MTT assay. A549 cells were incubated with TGF-β1 (10 ng/ml) with or without indicated concentrations of BYHWT for 24 h, 48 h and 72 h, respectively. Data are presented as mean ± SD, *n* = 5. *P<0.05, **P<0.01, VS. control; △*P*<0.05, △△*P*<0.01,VS. TGF-β1

To elucidate the mechanism for BYHWT in the treatment of PF, we utilized a common in vitro PF model, TGF-β1 induced A549 cells. Cell viability assay was first performed. A549 cells were treated with 10 ng/ml TGF-β1 with or without different concentrations of BYHWT for 24 h, 48 h and 72 h, respectively. As illustrated in Fig. 1, BYHWT can significantly inhibit the cell growth of TGF-β1 stimulated A549 cells.

### BYHWT suppressed TGF-β1-induced EMT and collagen I in pulmonary fibrosis in A549 cells

**Fig. 2:**
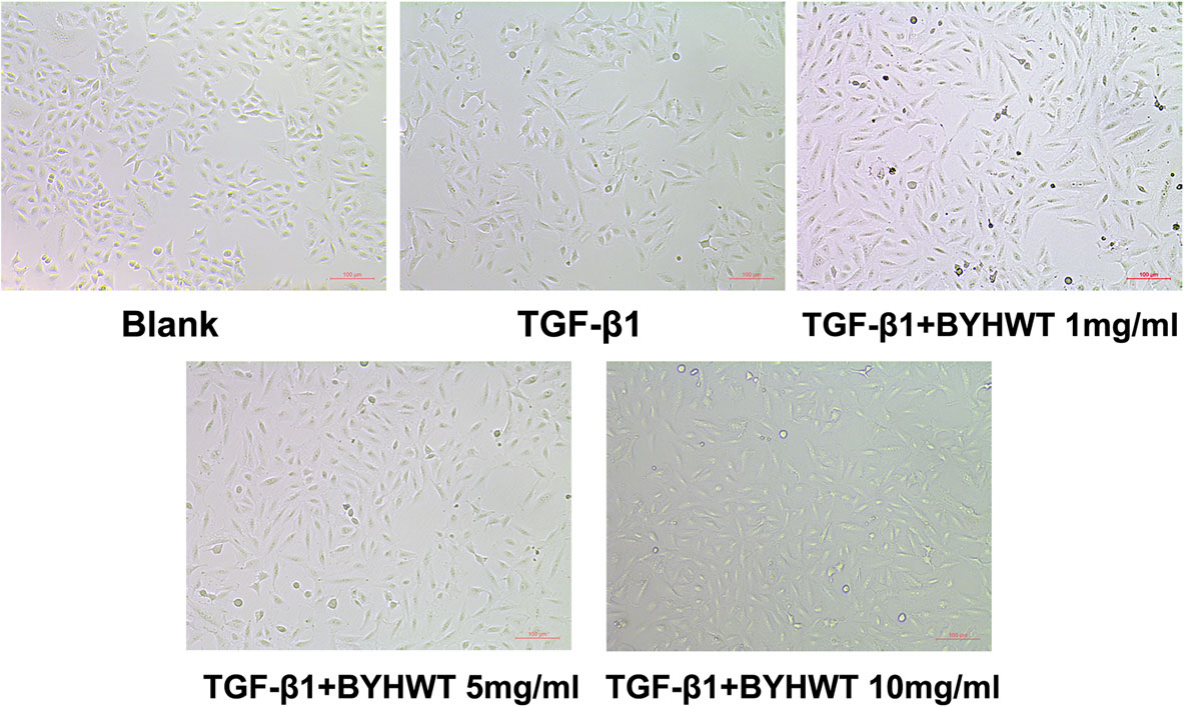
Effect of BYHWT on cell morphology in TGF-β1 treated A549 cells. A549 cells were incubated with TGF-β1 (10 ng/ml) with or without indicated concentrations of BYHWT for 24 h, followed by observation by a inverted phase contrast microscope (× 200 fold)

TGF-β1 mediated EMT and collagen accumulation plays an important role in the development of PF. To identify whether BYHWT can inhibit this process, we did a series of experiments. As presented in Fig. 2, following TGF-β1 treatment, A549 cells exhibited a spindle-like shape with mesenchymal morphology and were more isolated from each other. Interestingly, this change in morphology was reversed by co-treatment with BYHWT and TGF-β1.

**Fig. 3:**
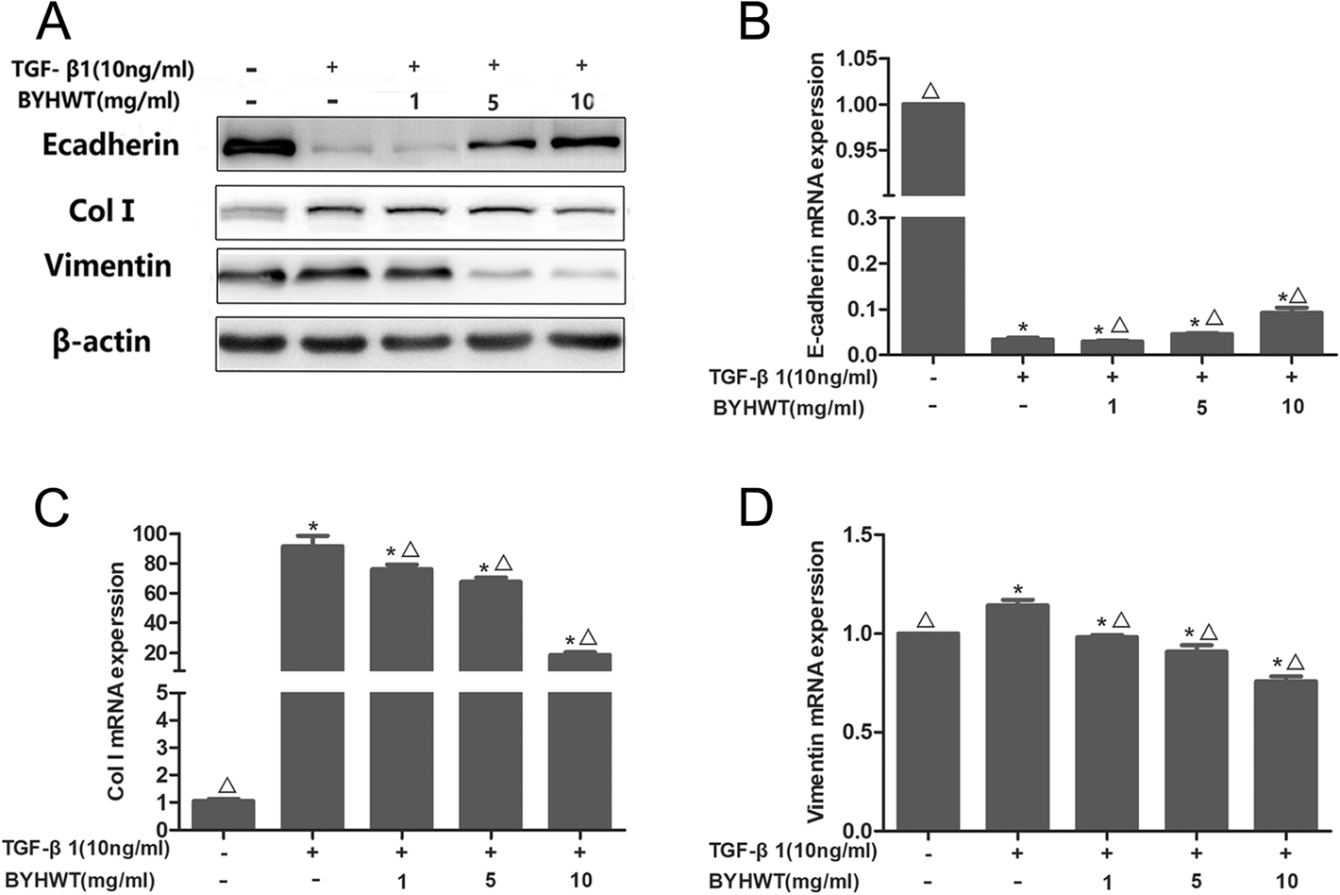
Inhibition of TGF-β-induced EMT and colleage accmulation by BYHWT in A549 cells. (**a**-**d**) A549 cells were incubated with TGF-β1 (10 ng/ml) in the absence or presence of indicated concentrations of BYHWT. Twenty-four hours later, the changes of mRNA of EMT markers (E-cadherin, Vimentin) and Col I were analyzed by RT-qPCR, while 72 h later, the changes of mRNA of EMT markers (E-cadherin, Vimentin) and Col I were analyzed by western blot. Data are presented as mean ± SD, *n* = 3. *P<0.05, VS. control; △*P*<0.05,VS. TGF-β1

**Fig. 4:**
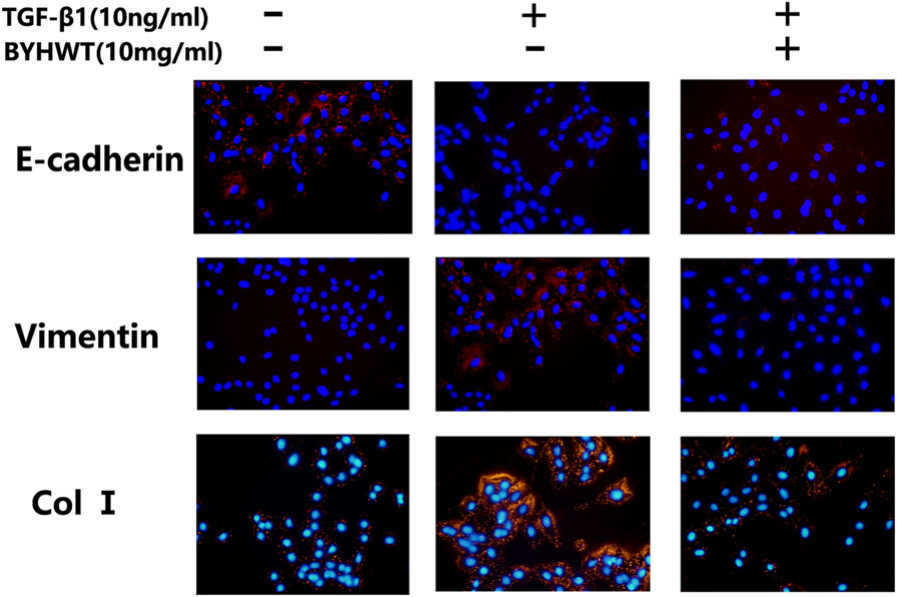
Effect of BYHWT on TGF-β induced EMT and colleage accmulation in A549 cells by immunofluorescence. A549 cells were incubated with TGF-β1 (10 ng/ml) in the absence or presence of BYHWT (10 mg/ml BYHWT) for 72 h. The nucleus were stained with DAPI and images were visualized by immunofluorescence microscopy (× 200 fold)

Next, we went on to characterize the expression of EMT-related markers. As expected, in TGF-β1 treated A549 cells, the mesenchymal marker Vimentin was increased, while the epithelial marker E-cadherin was reduced, at both protein (Fig. 3a) and mRNA (Fig. 3b,c,d) levels. As expected, BYHWT remarkably elevated E-cadherin and inhibited Vimentin in a dose-dependent manner. Moreover, the elevated protein and mRNA levels of ColI induced by TGF-β1 were significantly decreased by the administration of BYHWT. Consistent with the findings of Western Blot, our immunofluorescence staining results also demonstrated the effect of BYHWT in attenuating EMT and Col I accumulation (Fig. 4).

### BYHWT inhibited PI3K/Akt signaling pathway

**Fig. 5:**
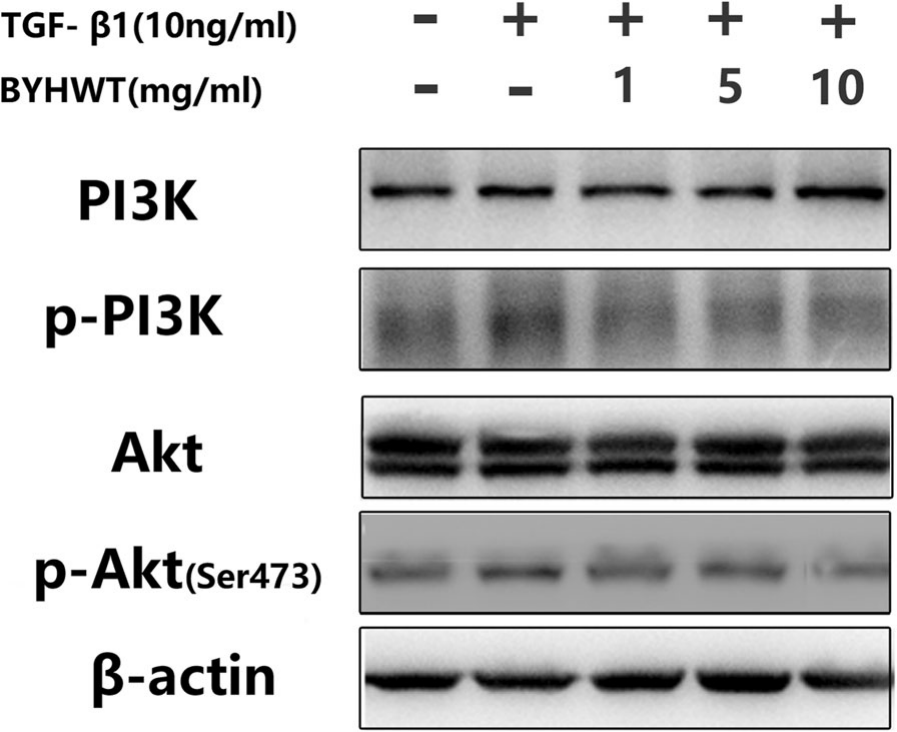
Inhibition of TGF-β-induced EMT by BYHWT was associated with the suppression of the PI3K/Akt pathway. A549 cells were incubated with TGF-β1 (10 ng/ml) in the absence or presence of BYHWT (1, 5 and 10 mg/ml) for 1 h. Cell lysates were collected and PI3K, p-PI3K, Akt and p-Akt were analyzed by western blot

To determine the mechanism how BYHWT inhibited EMT and Col I deposition, we examined the effect of BYHWT on PI3K/Akt signaling pathway, which has been demonstrated to play a major role in TGF-β-induced EMT. As illustrated in Fig. 5, p-PI3K and p-Akt in TGF-β1 treated cells were significantly higher than that in the control group, and BYHWT significantly inhibited the activation of p-PI3K and p-Akt in a dose-dependent manner.

**Fig. 6:**
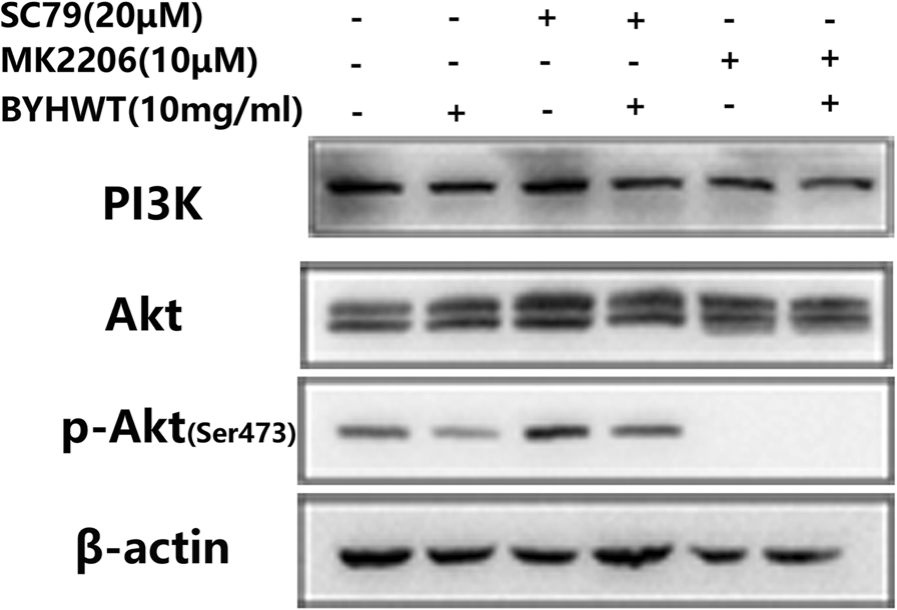
Effect of BYHWT on Akt activation in SC79-treated A549 cells. A549 cells were treated with SC79 (20 μM) or MK2206 (10 μM), with or without the administration of 10 mg/ml BYHWT for 1 h. Cell lysates were collected and followed by analysis of PI3K, Akt and p-Akt by western blot

**Fig. 7:**
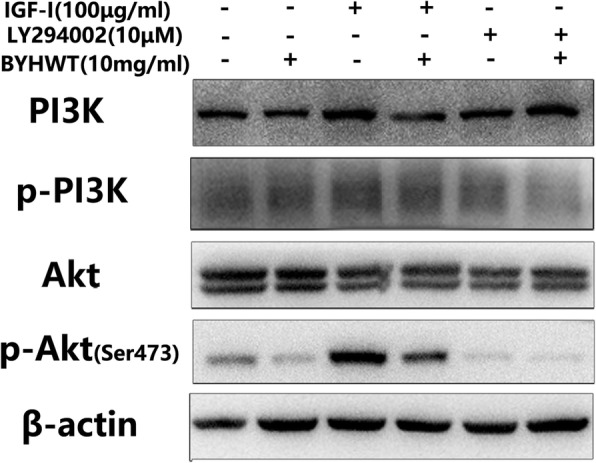
Effect of BYHWT on PI3K activation in IGF-I-treated A549 cells. A549 cells were treated with IGF-I (100 μg/ml) or LY294002 (10 μM), with or without the administration of 10 mg/ml BYHWT for 1 h. Cell lysates were collected and followed by analysis of PI3K, p-PI3K, Akt and p-Akt by western blot

With the utilization of PI3K/Akt pathway activators (IGF-I, SC79) and inhibitors (MK2206, LY294002), we further identified the exact targets of BYHWT on PI3K/Akt pathway. As shown in Fig. 6, SC79 significantly activated p-Akt, whereas the elevated level of p-Akt was sharply reduced by the co-administration of BYHWT. MK2206 inhibited the activation of p-Akt, and the co-treatment of BYHWT did not increase p-Akt. Furthermore, western blot analysis (Fig. 7) showed that p-PI3K and the down-streaming p-Akt were activated by IGF-I. After treatment by BYHWT, the level of p-PI3K and p-Akt protein were significantly lower than that in the IGF-1 group cells. In addition, p-PI3K and p-Akt was decreased by LY294002, but the co-treatment of BYHWT resulted in a further decrease.

## Discussion

PF is a severe inflammatory interstitial lung disease with poor prognosis, high mortality, and increasing incidence and prevalence [16]. However, the treatments of PF still remain not promising. In addition to lung transplantation, the progression and outcome of PF cannot be potently prevented by other special treatments [17]. Exploring novel agents to cure PF is still highly desired.

With the deep understanding of the pathogenesis of PF by traditional Chinese medicine theory, Chinese medicine has a prospect for the treatment of pulmonary fibrosis [18]. BYHWT, as a classical traditional Chinese medicine formula, was widely used for supplementing qi and activating blood circulation. It has shown that BYHWT can alleviate angiogenesis of rats by inhibiting the expression of VEGF, which is a key growth factor for pulmonary fibrosis [19, 20]. Moreover, a recent study showed that BYHWT can effectively reduce colI accumulation, alleviate inflammation of lung tissue and reduce serum hydroxyproline in bleomycin-induced PF rats, and this effect may be related to the down-regulation of Akt [21].

Human alveolar epithelial A549 cells stimulated by TGF-β1 has been frequently utilized as in vitro model of PF [22]. The results of cell morphology, western blot analysis, RT-qPCR and immunofluorescence indicated that PF cell model were successfully established in TGF-β1 induced of A549 cells. Based on the MTT assay results for cytotoxic effects of the co-treatment of TGF-β1 and BYHWT, A549 cells were treated with BYHWT at concentrations of 1, 5, and 10 mg/ml with the co-administration of TGF-β1. Results showed that BYHWT can lessen the mesenchymal morphology of A549 cells. Moreover, according to the results of RT-qPCR, western blotting and immunofluorescence staining, the addition of BYHWT significantly increased E-cadherin, as well as reduced Col I and Vimentin. EMT and collagen accumulation is activated by TGF-β1, characterized by the loss of epithelial characteristics (E-cadherin), the acquisition of a mesenchymal phenotype (Vimentin) and the increase of Col I [9]. Therefore, our results indicated that BYHWT can suppress TGF-β1 mediated EMT and TGF-β1 induced collagen deposition in PF.

In recent years, it has been demonstrated that activation of PI3K/Akt signaling pathway plays a key role in the development and progression of PF, which could promote fibroblasts proliferation and inhibit fibroblasts apoptosis [23]. Next, we set out to identify whether PI3K/Akt pathway is involved in inhibiting EMT and Col I accumulation by BYHWT. Results demonstrated that BYHWT remarkably suppressed p-PI3K and p-Akt, which was activated by TGF-β1, strongly indicating that the effect of inhibiting EMT and Col I accumulation in TGF-β1 induced A549 cells by BYHWT may related to the inhibition of PI3K/Akt signaling pathway.

Activation of p-Akt has been associated with Col I expression and EMT progress in alveolar epithelial cells [24]. Therefore, we studied the effect of BYHWT on the activation of p-Akt. SC79, a specific Akt activator and MK2206, a specific Akt inhibitor, were used to further identify the effect of BYHWT on Akt activation in PI3K/Akt signaling pathway [25, 26]. Results showed that the elevated level of p-Akt induced by SC79, was down-regulated by BYHWT, and p-Akt was remarkably suppressed by the co-treatment of BYHWT and MK2206, indicating that BYHWT can directly inhibit the activation of Akt.

PI3K is a kinase upstream of Akt, which regulates down-streaming effects of EMT progress by affecting p-Akt [27]. Additionally, Akt is an effector kinase downstream from PI3K [28], whether BYHWT blocks the PI3K/Akt signaling pathway through acting on the target of PI3K remains unclear and requires further investigation. Therefore, we used a specific PI3K activator, IGF-1 and a PI3K inhibitor, LY294002, to further identify whether BYHWT can act on PI3K [29]. Results showed that PI3K and Akt were strongly phosphorylated by IGF-1, while BYHWT inhibited the activation of those proteins. Moreover, p-Akt and p-PI3K were significantly inhibited by the co-treatment of BYHWT and LY294002, demonstrating that BYHWT can also directly suppress the activation of PI3K.

In conclusion, we found that a classical Chinese medicine formula BYHWT can inhibit TGF-β1 induced EMT and collagen accumulation in a in vitro model of PF via suppressing PI3K/Akt signaling pathway. Moreover, BYHWT can not only inhibit the activation of PI3K, resulting in the down-regulation of the down streaming Akt, but also inhibit the activation of Akt directly. Thus, BYHWT could be considered as a potential therapeutic strategy for the treatment of PF.

## Conclusions

BYHWT has been frequently used in the treatment of PF in China, however the mechanism remains largely unknown. With the utilization of in vitro model of PF, we found BYHWT can inhibit TGF-β1 induced EMT and collagen accumulation through suppressing PI3K/Akt signaling pathway. BYHWT can be adopted in clinical settings as a part of alternative medicine for PF patients and more extensive investigation of their mechanisms and evidence-based knowledge is still required.

## Data Availability

The datasets analyzed during the current study are available from the corresponding author on reasonable request.

## Abbreviations

AECs: Alveolar epithelial cells
BCA: Bicinchoninic acid
BYHWT: Buyang Huanwu Tang
DAPI): 4′, 6-diamidino-2-phenylindole
DMEM: Dulbecco’s Modified Eagle’s Medium
EMT: Epithelial-to-mesenchymal transition
FBS: Fetal bovine serum
IGF-1: Insulin-like growth factor-1
MTT: 3-(4,5-dimethylthizaol-2-yl)-2,5-diphenyl tetrazolium bromide
RT-qPCR: Real-time quantitative polymerase chain reaction
TGF-β: Transforming growth factor-β

## References

[1] Zhou XM, Wen GY, Zhao Y, Liu YM, Li JX (2013). Inhibitory effects of alkaline extract of citrus reticulata on pulmonary fibrosis. J Ethnopharmacol.

[2] Mojiri-Forushani H, Hemmati AA, Dehghani MA, Malayeri AR, Pour HH (2017). Effects of herbal extracts and compounds and pharmacological agents on pulmonary fibrosis in animal models: a review. Chin J Integr Med.

[3] Hopkins RB, Burke N, Fell C, Dion G, Kolb M (2016). Epidemiology and survival of idiopathic pulmonary fibrosis from national data in Canada. Eur Respir J.

[4] Li L, Huang W, Li K, Zhang K, Lin C, Han R, Lu C, Wang Y, Chen H, Sun F (2015). Metformin attenuates gefitinib-induced exacerbation of pulmonary fibrosis by inhibition of TGF-beta signaling pathway. Oncotarget..

[5] Mojiri-Forushani H, Hemmati AA, Khodadadi A, Rashno M (2018). Valsartan attenuates bleomycin-induced pulmonary fibrosis by inhibition of NF-kappaB expression and regulation of Th1/Th2 cytokines. Immunopharmacol Immunotoxicol.

[6] Wang P, Wang Y, Nie X, Braini C, Bai R, Chen C (2015). Multiwall carbon nanotubes directly promote fibroblast-myofibroblast and epithelial-mesenchymal transitions through the activation of the TGF-beta/Smad signaling pathway. Small.

[7] Takata T, Motoo Y, Tomosugi N (2014). Effect of Saikokeishito, a Kampo medicine, on hydrogen peroxide-induced premature senescence of normal human dermal fibroblasts. J Integr Med.

[8] Lofdahl A, Rydell-Tormanen K, Larsson-Callerfelt AK, Wenglen C, Westergren-Thorsson G (2018). Pulmonary fibrosis in vivo displays increased p21 expression reduced by 5-HT2B receptor antagonists in vitro - a potential pathway affecting proliferation. Sci Rep.

[9] Yi GZ, Liu YW, Xiang W, Wang H, Chen ZY, Xie SD, Qi ST (2016). Akt and beta-catenin contribute to TMZ resistance and EMT of MGMT negative malignant glioma cell line. J Neurol Sci.

[10] Zhao H, Wu QQ, Cao LF, Qing HY, Zhang C, Chen YH, Wang H, Liu RY, Xu DX (2014). Melatonin inhibits endoplasmic reticulum stress and epithelial-mesenchymal transition during bleomycin-induced pulmonary fibrosis in mice. PLoS One.

[11] Xu X, Dai H, Geng J, Wan X, Huang X, Li F, Jiang D, Wang C (2015). Rapamycin increases CCN2 expression of lung fibroblasts via phosphoinositide 3-kinase. Lab Investig.

[12] Liu Q, Chu H, Ma Y, Wu T, Qian F, Ren X, Tu W, Zhou X, Jin L, Wu W (2016). Salvianolic acid B attenuates experimental pulmonary fibrosis through inhibition of the TGF-beta signaling pathway. Sci Rep.

[13] Zhang XL, Xing RG, Chen L, Liu CR, Miao ZG (2016). PI3K/Akt signaling is involved in the pathogenesis of bleomycininduced pulmonary fibrosis via regulation of epithelialmesenchymal transition. Mol Med Rep.

[14] Shaw LH, Chen WM, Tsai TH (2013). Identification of multiple ingredients for a Traditional Chinese Medicine preparation (bu-yang-huan-wu-tang) by liquid chromatography coupled with tandem mass spectrometry. Molecules.

[15] Yun Z, Liguo C, Mingzhi X, Zhen J, University J (2019). Research progress of Buyang Huanwu decoction in the treatment of pulmonary fibrosis. Clin J Tradit Chin Med.

[16] Aiello M, Bertorelli G, Bocchino M, Chetta A, Fiore-Donati A, Fois A, Marinari S, Oggionni T, Polla B, Rosi E (2017). The earlier, the better: impact of early diagnosis on clinical outcome in idiopathic pulmonary fibrosis. Pulm Pharmacol Ther.

[17] Jones MG, Richeldi L (2016). Recent advances and future needs in interstitial lung diseases. Semin Respir Crit Care Med.

[18] Li LC, Kan LD (2017). Traditional Chinese medicine for pulmonary fibrosis therapy: Progress and future prospects. J Ethnopharmacol.

[19] Xu JN, Que HF, Tang HJ (2009). Effects and action mechanisms of Buyang Huanwu decoction in wound healing of chronic skin ulcers of rats. J Chin Integr Med.

[20] Ebina M (2017). Pathognomonic remodeling of blood and lymphatic capillaries in idiopathic pulmonary fibrosis. Respir Investig.

[21] Wang X, Li X, Wang LN, Pan JJ, Yang X, Wei YL (2018). Buyang Huanwu decoction ameliorates bleomycin-induced pulmonary fibrosis in rats via downregulation of related protein and gene expression. Evid Based Complement Alternat Med.

[22] Baek SH, Ko JH, Lee JH, Kim C, Lee H, Nam D, Lee J, Lee SG, Yang WM, Um JY (2017). Ginkgolic acid inhibits invasion and migration and TGF-beta-induced EMT of lung cancer cells through PI3K/Akt/mTOR inactivation. J Cell Physiol.

[23] Yan W, Xiaoli L, Guoliang A, Zhonghui Z, Di L, Ximeng L, Piye N, Li C, Lin T (2016). SB203580 inhibits epithelial-mesenchymal transition and pulmonary fibrosis in a rat silicosis model. Toxicol Lett.

[24] Conte E, Fruciano M, Fagone E, Gili E, Caraci F, Iemmolo M, Crimi N, Vancheri C (2011). Inhibition of PI3K prevents the proliferation and differentiation of human lung fibroblasts into myofibroblasts: the role of class I P110 isoforms. PLoS One.

[25] Zheng K, Zhang Q, Lin G, Li Y, Sheng Z, Wang J, Chen L, Lu HH (2017). Activation of Akt by SC79 protects myocardiocytes from oxygen and glucose deprivation (OGD)/re-oxygenation. Oncotarget..

[26] Xiang RF, Wang Y, Zhang N, Xu WB, Cao Y, Tong J, Li JM, Wu YL, Yan H (2017). MK2206 enhances the cytocidal effects of bufalin in multiple myeloma by inhibiting the AKT/mTOR pathway. Cell Death Dis.

[27] Le Cras TD, Korfhagen TR, Davidson C, Schmidt S, Fenchel M, Ikegami M, Whitsett JA, Hardie WD (2010). Inhibition of PI3K by PX-866 prevents transforming growth factor-alpha-induced pulmonary fibrosis. Am J Pathol.

[28] He LF, Xu HW, Chen M, Xian ZR, Wen XF, Chen MN, Du CW, Huang WH, Wu JD, Zhang GJ (2017). Activated-PAK4 predicts worse prognosis in breast cancer and promotes tumorigenesis through activation of PI3K/AKT signaling. Oncotarget..

[29] Xu L, He S, Yin P, Li D, Mei C, Yu X, Shi Y, Jiang L, Liu F (2016). Punicalagin induces Nrf2 translocation and HO-1 expression via PI3K/Akt, protecting rat intestinal epithelial cells from oxidative stress. Int J Hyperth.

